# Peri-operative management of hysterostomy in a parturient with complete heart block, placenta accreta and intrauterine death

**DOI:** 10.1186/1471-2253-14-49

**Published:** 2014-06-28

**Authors:** Vineya Rai, Ina I Shariffuddin, Yoo K Chan, Rajesh K Muniandy, Kang K Wong, Sukcharanjit Singh

**Affiliations:** 1 Department of Anaesthesiology, Faculty of Medicine, University of Malaya, 50603 Kuala Lumpur, Malaysia; 2 Department of Medicine Based, School of Medicine, Universiti Malaysia Sabah, Kota Kinabalu, Sabah, Malaysia

**Keywords:** Complete heart block, Pregnancy, Hysterostomy, Hysterectomy

## Abstract

**Background:**

Complete heart block in pregnancy has serious implications particularly during the period of delivery. This is more so if the delivery is an operative one as the presence of heart block may produce haemodynamic instability in the intra operative period. We report a unique case of a pregnant mother with complete heart block undergoing hysterostomy, complicated by placenta accreta and intrauterine death.

**Case presentation:**

A 37 year old Malaysian Chinese parturient was admitted at 25 weeks gestation following a scan which suggested intrauterine death and placenta accreta. She was diagnosed to have congenital complete heart block after her first delivery eight years previously but a pacemaker was never inserted. These medical conditions make her extremely likely to experience massive bleeding and haemodynamic instability. Among the measures taken to optimise her pre-operatively were the insertion of a temporary intravenous pacemaker and embolization of the uterine arteries to minimize peri-operative blood loss. She successfully underwent surgery under general anesthesia, which was relatively uneventful and was discharged well on the fourth post-operative day.

**Conclusion:**

Congenital heart block in pregnancies in the presence of potential massive bleeding is best managed by a team, with meticulous pre-operative optimization. Suggested strategies would include insertion of a temporary pacemaker and embolization of the uterine arteries to reduce the risk of the patient getting into life threatening situations.

## Background

Complete heart block (CHB) in pregnancy is not a common encounter [1]. However it has serious implications in pregnancy particularly during the period of delivery. This is more so if the delivery is an operative one as the presence of heart block may not allow the mother to respond well to haemodynamic instability. There are reports of cases of congenital complete heart block in the literature where mothers have been paced temporarily but there are equally others where no pacemakers were used. Those who are managed without pacemakers risk having complications, including death.

We report the successful management of a parturient with a unique set of problems besides just congenital CHB. She had placenta accreta and intrauterine death needing hysterostomy which put her at extreme risk of massive bleeding and haemodynamic instability. The strategies for safe management included the prophylactic insertion of a temporary intravenous pacemaker and the pre-emptive embolization of the uterine arteries which has been shown in other studies to minimize blood loss perioperatively [2].

## Case presentation

**Figure 1:**
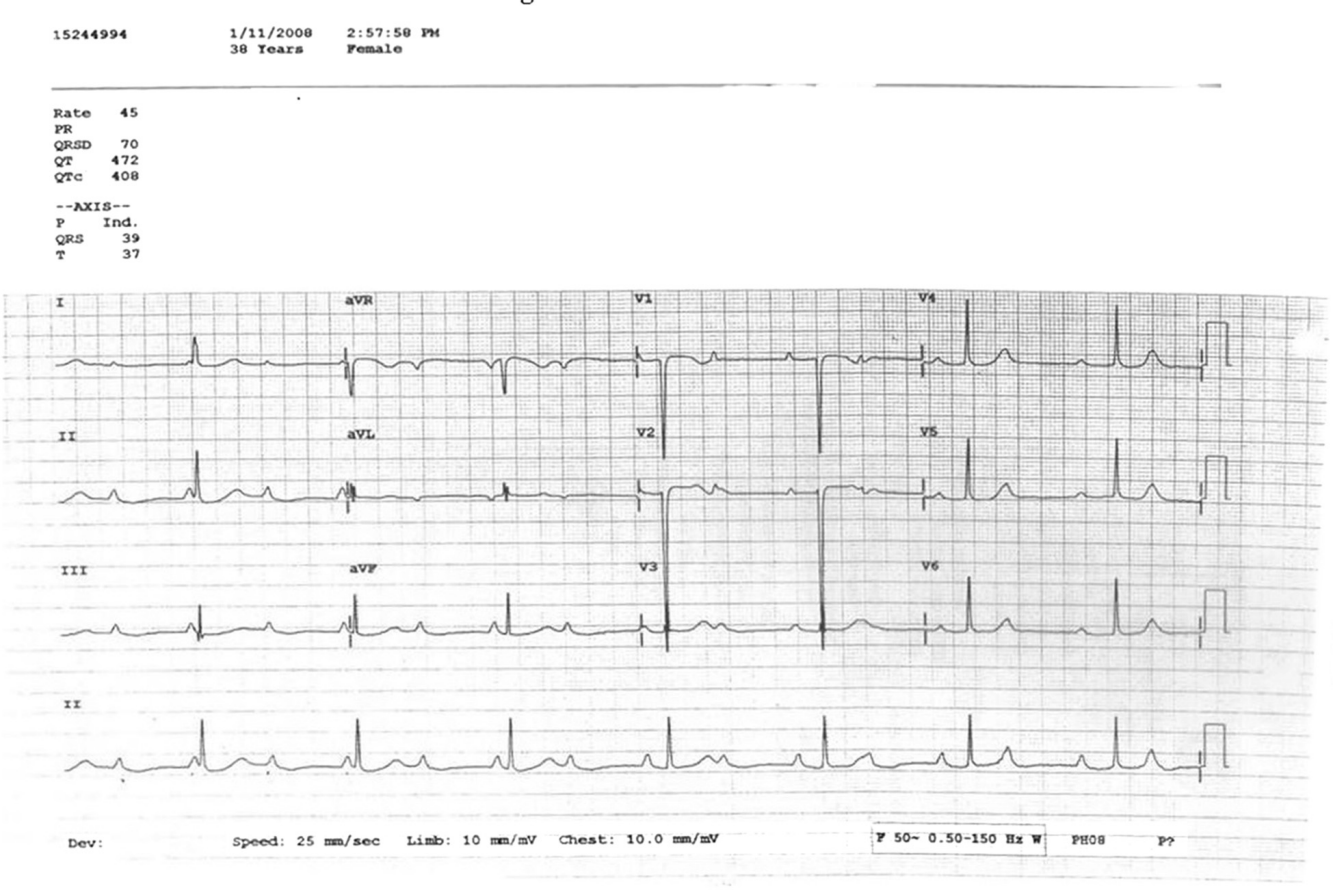
Patient’s ECG.

The patient, a 37 year old Malaysian Chinese parturient (Gravida 4 Para 2 + 1) was admitted at 25 weeks gestation following a routine scan which suggested intrauterine death. A repeat scan done when she had per vaginal bleeding confirmed the death and showed a grade 3–4 placenta praevia with suggestions of a placenta accreta.Her two previous deliveries were via caesarean section for breech delivery 8 and 6 years prior to the current event in another hospital. She was confirmed to have a congenital complete heart block after her first delivery and was followed up in the National Heart Insitute where she was treated conservatively. Throughout the period of current and previous pregnancies, she has never been symptomatic of CHB; chest pain, palpitations or syncopal attacks. Her ECG showed a complete heart block with narrow QRS complex, indicating a high nodal impulse with a rate of 50/min (Figure 1). Her blood pressure was 140/80 mmHg. Bedside echocardiogram was relatively normal except for mild mitral regurgitation. Her serum thyroid stimulating hormone and antinuclear antibody levels were normal. Coagulation profile showed a slightly elevated prothrombin time of 1.24 s and an international normalized ratio of 1.3. Her fibrinogen levels were normal.

The obstetrician opted for hysterostomy to deliver the baby with an alternative plan of performing a caesarean hysterectomy in the event of uncontrolled bleeding. Prior to the surgery, bilateral uterine artery embolization was performed by a radiologist. A temporary intravenous pacemaker was also inserted by a cardiologist.

**Figure 2:**
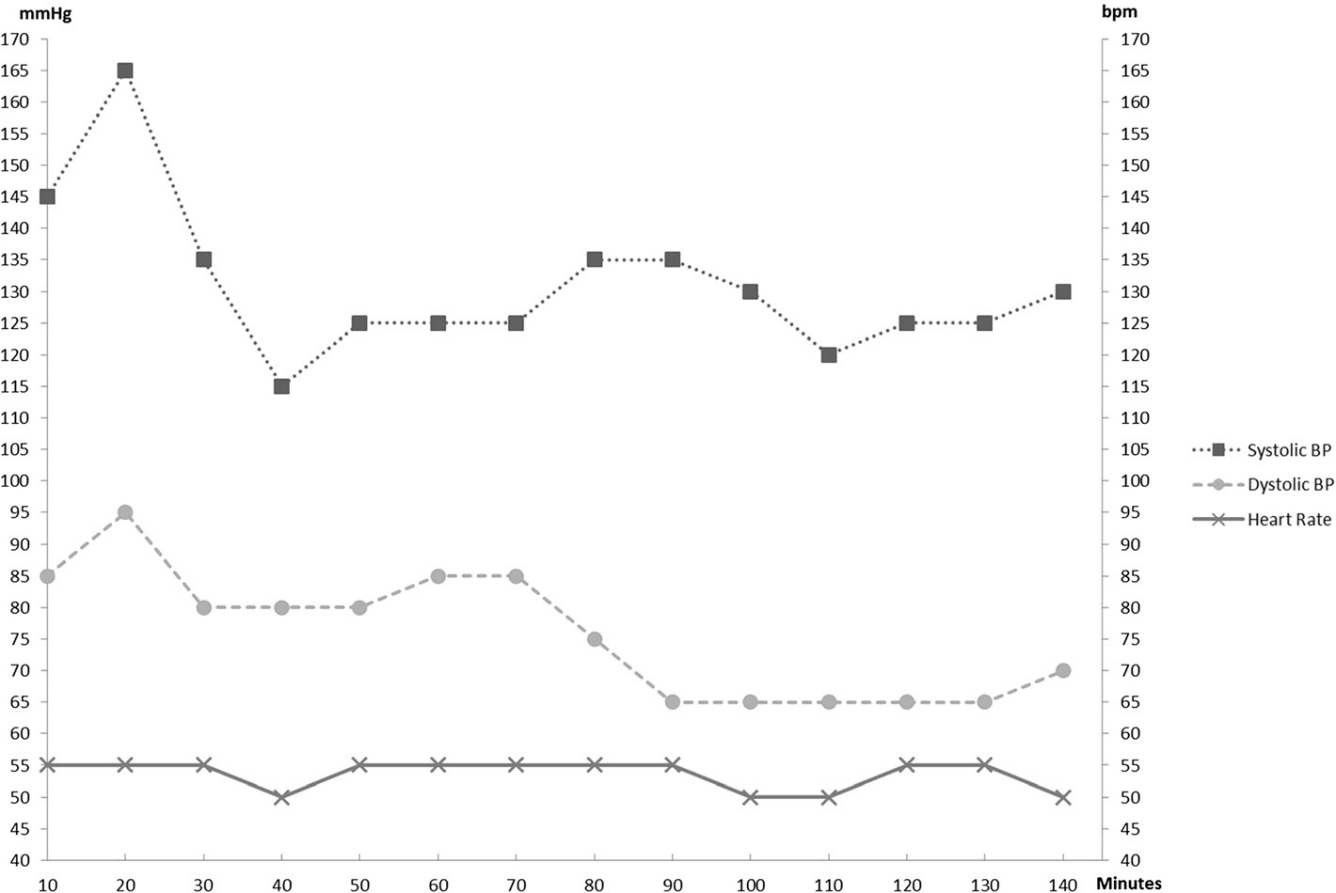
Patient’s peri-operative blood pressure and heart rate.

General anesthesia was induced with fentanyl 100 μg, etomidate 18 mg and suxamethonium 100 mg. Anaesthesia was maintained with 2% sevoflurane in oxygen/air 50%. Pain relief was with morphine boluses and continued muscle relaxation with rocuronium. Tranexamic 1 g was also infused intra-operatively.Patient’s heart rate was monitored and averaged between 50–60 beats/minute which did not require pacemaker activation. Intra-arterial blood pressure monitoring did not show any instability. The patient’s haemodynamics remained stable intra-operatively (Figure 2).

The total blood loss was 1.2 L and patient was transfused 3 units of packed cells intra-operatively. The pre-operative haemoglobin was 11.3 g/dL and the immediate post-operative value was 7.9 g/dL. A total abdominal hysterectomy was done to control the bleeding.

Post-operatively, the patient was extubated as she remained stable hemodynamically. She was monitored in the intensive care unit and the pacemaker was removed 48 hours later as she remained stable hemodynamically. Patient was discharged home on the 4th post-operative day.

## Discussion

Complete heart block, also known as third degree atrioventricular (AV) or simply third degree heart block is a disorder of the cardiac conduction system with complete absence of AV conduction. Aetiologically, CHB can be divided into two groups: congenital complete heart block and acquired complete heart block (ACHB) [3]. Congenital CHB in pregnancy is associated with connective tissue disease but this was not present in our patient. Most cases reported in the literature suggest that uncomplicated brady-arrhythmia during pregnancy in the absence of significant underlying heart disease, results in a favourable outcome for both mother and baby. In addition, uncomplicated brady-arrhythmias including asymptomatic CHB has not been reported to cause any maternal death [4]. However, our patient also had additional risk factors mainly massive bleeding secondary to placenta accreta and potential disseminated intravascular coagulation. Thus, our patient is unique in that when faced with massive bleeding, she potentially would not be able to mount an adequate heart rate response due to the CHB. In light of that, a temporary pacemaker was inserted to cope with this likely possibility.

In the 1950s, Epstein and Altman suggested that without pacemaker insertion, pregnancy in women with complete heart block was associated with high maternal and fetal mortality [5]. However, subsequent case series reported experiences without the insertion of pacemaker in uncomplicated pregnancies and deliveries [6]. These deliveries however were not at risk of obstetric haemorrhage. While our patient did not at any juncture use the temporary pacemaker, we felt that the insertion was fully justified to cover for the eventuality of a life-threatening situation.

The team took measures to reduce the risk of haemorrhage by ensuring that she had no coagulation abnormality despite the intra-uterine death. Bilateral uterine artery embolization was performed in this patient before the fetus was delivered as it was already confirmed dead. This procedure does not compromise long term uterine integrity [7]. Traditional methods of controlling obstetric haemorrhage are by bilateral uterine artery or hypogastric artery ligation. These traditional methods produce low success rates in bleeding reduction due to the presence of extensive collateral circulation in the pelvis and has been estimated to be as low as 42% [8]. This is in comparison to embolization which has a success rate of more than 90% [9]. In addition, if a caesarean hysterectomy is needed, embolization can slow the rate of blood loss hence improving the surgical field. Caesarean hysterectomy in the presence of severe haemorrhage is an extremely challenging procedure which carries a high risk of iatrogenic damage to other pelvic structures especially ureters and bladder [10]. Slowing the rate of blood loss improves the surgical field, making the procedure technically easier thus reducing the risk of surgical complications [11]. In the presence of a significant blood loss in our patient, the team felt it was safer to proceed with hysterectomy to reduce the risk of post partum bleeding.

In patients at risk of major haemorrhage, choosing the best form of anaesthesia can be challenging. The advantage of regional anaesthesia is that it omits the use of volatile agents, which may contribute to uterine atony. Regional anaesthesia has also been shown to reduce transfusion requirements [12-14]. We chose general anaesthesia over regional anaesthesia as our patient had a definite risk of major obstetric haemorrhage complicated with CHB. With general anaesthesia, we had a better haemodynamic control and could focus on keeping up with the blood loss correction. Furthermore, the risk of conversion from regional to general anaesthesia at the time of torrential haemorrhage may add to further cardiovascular instability.

Etomidate was chosen over thiopentone as our induction agent as the former has been shown to be superior in maintaining cardiovascular stability. With the pacemaker in place we felt confident about using fentanyl, etomidate and suxamethonium, the combination of which can potentially cause bradycardia [15]. Our patient did not suffer this complication during the induction process.

## Conclusion

In summary, congenital CHB in pregnancies in the presence of potential massive bleeding is best managed as a team, with meticulous pre-operative optimization. Suggested strategies would include insertion of a temporary pacemaker and embolization of the uterine arteries in situations where excessive bleeding is likely to contribute to a life threatening situation.

## Consent

Written informed consent was obtained from the patient for publication of this case report. A copy of the written consent is available for review by the Editor of this journal.

## Abbreviations

ECG: Electrocardiogram; INR: International normalized ratio; CHB: Complete heart block; ACHB: Acquired complete heart block; AV: Atrioventricular.

## Competing interests

The authors declare that they have no competing interests.

## Authors’ contributions

VR: Preparation of the manuscript and involvement in the case. IIS: Anesthesiologist involved in the case. YKC: Conceived of the study, and participated in its design and coordination and helped to draft the manuscript. RKM: Preparation of the manuscript. KKW: Preparation of the manuscript. SS: Preparation of the manuscript. All authors read and approved the final manuscript.

## Pre-publication history

The pre-publication history for this paper can be accessed here:

http://www.biomedcentral.com/1471-2253/14/49/prepub
